# Immune responses of urban firefighters following work in the heat

**DOI:** 10.1186/2046-7648-4-S1-A107

**Published:** 2015-09-14

**Authors:** Anthony Walker, Matthew Driller, Christos Argus, Ben Rattray

**Affiliations:** 1 University of Canberra, Research Institute for Sport and Exercise, University of Canberra, Canberra, Australia

## Introduction

When firefighters work in hot environments, immune responses can be elevated for up to 90 minutes [1-3], possibly increasing the likelihood of thrombotic events or illness [4]. Australian firefighters complete multi-day deployments following natural disasters. However, the extent of immune changes following extended intervals, particularly after an overnight rest, is poorly understood. Thus, this study aimed to assess changes in immune responses of urban firefighters up to 24 hours after a work bout in the heat.

## Methods

Forty-two male urban firefighters completed two twenty minute search tasks in a purpose built heat chamber (mean (SD) 100 (5 °C). Based on standard operating procedures for an Australian fire service, participants had a ten minute passive recovery outside the heat chamber between work bouts, where they consumed 600 mL of water. Core temperatures (T_c_) and heart rates (HR), along with platelet and leukocyte numbers were evaluated pre- and post-work and also following one and twenty-four hours of rest.

## Results

**Figure 1:**
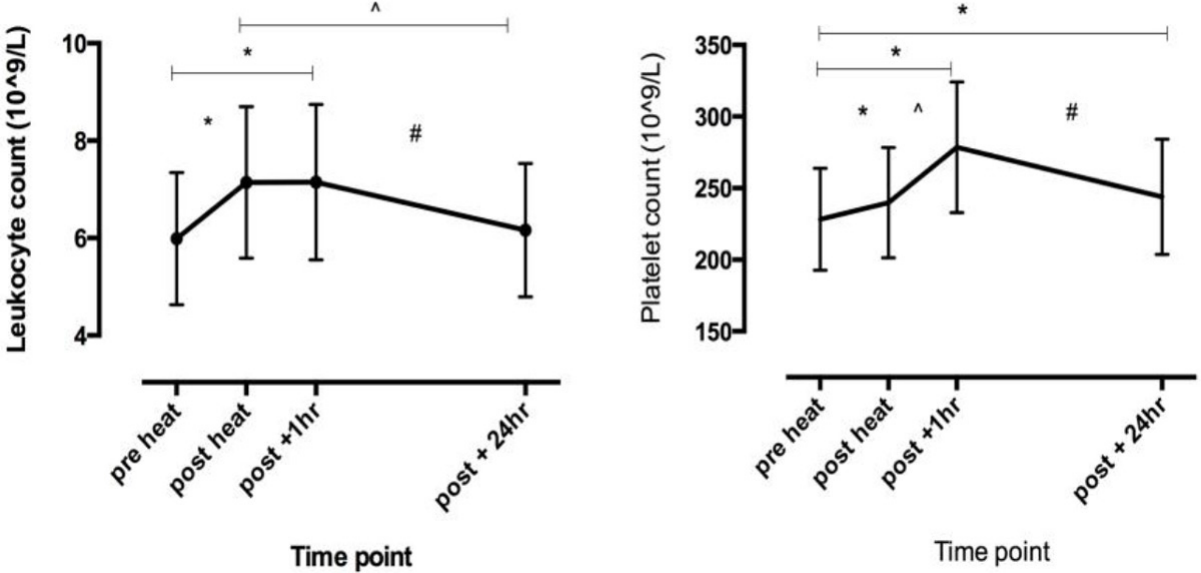
**Leukocyte and platelet numbers of firefighters during simulated work periods along with 1 and 24 hours post**. * Represents sig diff (p < 0.05) compared with pre-heat, ^ compared with post-heat and # compared with post + 1hr.

Increases in T_c _(+1.4 (0.5) °C, p < 0.01) and high HRs (90.9 (7.1) % HR_max_, p < 0.01)_, _were observed following the second work bout. Leukocyte and platelet numbers were significantly increased (p < 0.01) post-work, with platelets continuing to increase during one hour of passive recovery (+31.2 (31.3) 10^9 ^L, p < 0.01) (Figure 1). Further, platelets remained elevated 24 hours later (+15.9 (19.6) 10^9^L, p < 0.01).

## Discussion

Increases in temperatures, HR and immune responses of participants directly following work in the heat reflect previous studies. However, this study is unique in demonstrating significantly elevated platelet numbers after a 24 hour period of rest. Any residual elevations in platelet numbers after extended rest may be increasing the risk of thrombotic events when firefighters work over multiple days in adverse environmental conditions.

## Conclusion

The ongoing changes to platelet numbers in the present study likely represent a significant factor in ensuring the health of firefighters during multi-day deployments. It is likely that changes in work practices and rehabilitation protocols can minimise changes to immune responses during multi-day events, particularly in hot regions.
